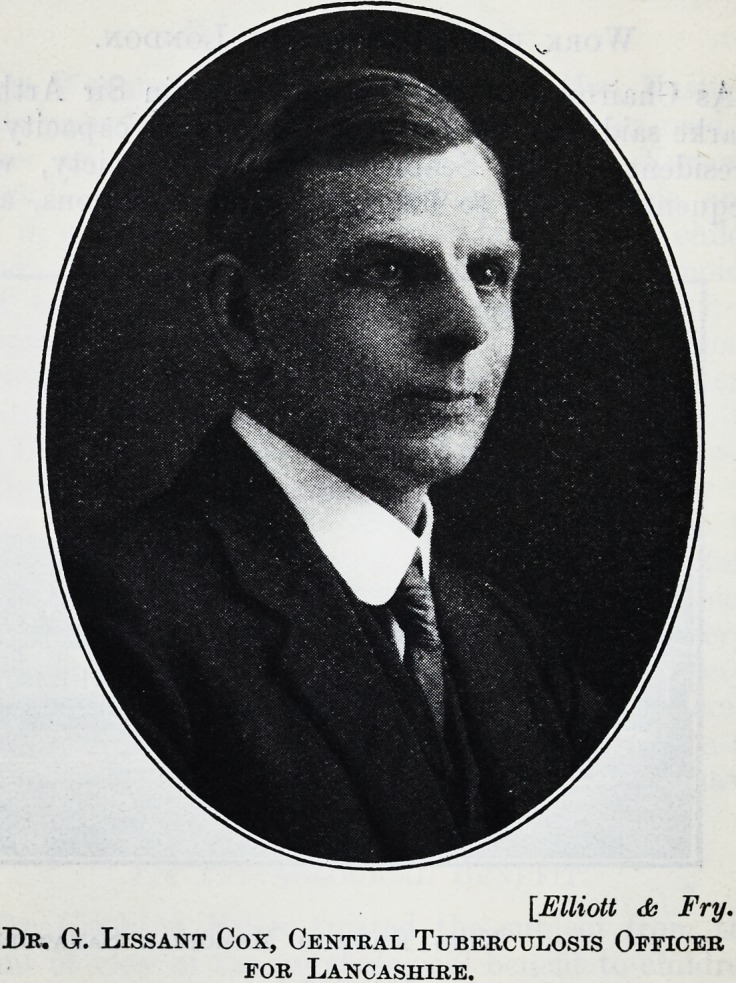# The Public Health: Interviews with Local Authorities: Tuberculosis in Lancashire

**Published:** 1924-08

**Authors:** 


### THE PUBLIC HEALTH.

### INTERVIEWS WITH LOCAL AUTHORITIES.

### XVIII.—FIGHTING TUBERCULOSIS IN LANCASHIRE.

It is stimulating to listen to Dr. Lissant Cox as he talks about his work as Central Tuberculosis Officer under the Lancashire County Council. So large a unit of administration is the county, and so impressed are the Council with the need for concentrating a section of their forces on fighting tuberculosis in their midst, that we find a County Tuberculosis Committee, separate and distinct from the general Health Committee of the county, with its own Chairman and its own Chief Medical Officer and staff. Be he never so skilled and zealous, the Tuberculosis Officer realises that he is as sounding brass and tinkling cymbal if he has not behind him an enthusiastic Committee and Chairman. Dr. Lissant Cox at the outset paid tribute to the support of his Committee and its Chairman, Mr. P. J. Hibbert, and the Vice-Chairman, Col. Charles Trimble, C.B., who were unavoidably absent from our interview.

[*Lafayette.*

Mr. P. J. Hibbert, Chairman of the Tuberculosis Committee of the Lancashire County Council.

[*Elliott & Fry.*

Dr. G. Lissant Cox, Central Tuberculosis Officer for Lancashire.

A Wide Field of Observation.

It is not claimed for the Lancashire scheme that it is complete. But it is comprehensive, it is progressive, and it is a big piece of work. The scheme, which embraces the prevention and treatment of tuberculosis, covers the whole of the Administrative County, containing an estimated population of 1,775,000, spread over a large geographical area (which excludes all the County Boroughs). There is thus a wide field of observation. Special treatment is provided for all forms of tuberculosis occurring in children and adults. The qualification for treatment is residence in the county, and treatment is provided free of cost. The cost of the scheme for 1924–5 is budgeted for at £155,084, of which £59,905, corresponding to a rate of 1.34 pence in the pound, will fall on the county rates, the balance being met by grants and other payments from the Government. The number of tuberculous persons under the supervision of the dispensary staff on January 1, 1924, was 11,000. This gives an incidence of 6.2 cases per 1,000 of the population. The death-rate from tuberculosis in the county for 1923 was easily the lowest on record. The keynote of the scheme is the dispensary organisation. A scheme fails on its most important side if this organisation is found wanting. The considerable prominence given to the more picturesque sanatorium side of tuberculosis work tends to obscure the work carried out through the dispensary organisation, which is the more important because its whole basis is towards early diagnosis and prevention rather than cure. The dispensary tuberculosis officer and his staff have to fulfil the most delicate duties, working in the closest co-operation with the medical practitioners and the local health officials, and effectively supervising the home treatment of the patients. The greater part of the county is divided into five large dispensary areas, each containing one or more branch dispensaries as well as the chief dispensary. Each area is under the charge of a consultant tuberculosis officer of high status and ability. To assist these officers there are eight assistant tuberculosis officers and thirty health visitors.

Home Supervision.

Ordinary symptomatic treatment is not undertaken at the dispensary if the patient is being attended by his own doctor ; the tuberculosis officer and his staff deal more particularly with the diagnosis of patients, having at their disposal special facilities for such work as bacteriological examination of specimens of sputum, X-ray examinations, blood tests, examination of urine, and observation of doubtful or difficult cases in suitable hospitals. There is an X-ray apparatus in the chief dispensary of each area, worked by the tuberculosis officer himself. At home patients are treated by their own doctors. The dispensary staff keep in touch with the home treatment of patients, and special attention is given to general hygienic and preventive measures carried out in close co-operation with the medical attendant and the local sanitary authority. The tuberculous patients are examined by the tuberculosis officers at suitable intervals. The thirty health visitors pay visits to the homes of the patients and assist at the dispensary clinics. They report on the conditions of the homes, and a copy of their report is sent to the local medical officer of health, whose attention is drawn to any sanitary defect. These nurses co-operate most effectively with the local sanitary authorities.

### Sleeping Conditions.

Of the total number of 1,731 pulmonary cases considered infectious or contagious at the end of 1922, 87 per cent. of the patients occupied a separate bed or bedroom ; patients without a separate bed were 13 per cent. of the total. The County Council have supplied a stock of bedsteads, mattresses, and nursing requisites, and these are lent out to necessitous cases in order to assist in the isolation of patients. Sleeping shelters are also supplied in suitable cases.

### The Unwilling Contact.

The drugging of patients finds little place in Dr. Lissant Cox's programme. Milk, cod liver oil and malt, in necessitous cases, yes. As regards drugs generally, the amount spent is negligible. Paper handkerchiefs, destroyed in the kitchen fire, said Dr. Cox, are cheaper and far more effective—a statement containing much food for reflection. Systematic examinations are made, especially among contacts of patients with positive sputum, in order to detect early or unsuspected cases of tuberculosis. Owing to indifference or unwillingness, considerable difficulty is often experienced in persuading contacts to present themselves for examination or even to submit themselves for examination at all, and the tuberculosis officer is therefore obliged to see the majority of them at their homes. The number of contacts examined in 1922 amounted to 1,626, revealing 79 definite cases of tuberculosis, equal to 48 per 1,000 contacts. This represents a much higher figure than any random sampling of the ordinary population would give.

### The Willing G.P.

If the contact is unwilling or slow to realise the value of the dispensary organisation, not so the better informed general practitioner. It is essential that he should refer to the tuberculosis officer cases while they are still doubtful. If special treatment is needed, it must be begun while the prospects of arrest of the disease are good. The success of the scheme so far as dispensary treatment is concerned cannot, by its very preventive aims, be measured by statistics. It can be measured to a large extent by the degree of confidence in the work shown by general practitioners, and Dr. Lissant Cox pointed with pardonable pride to the fact that no less than 77 per cent. of new cases are referred to the tuberculosis officers by the practitioners prior to the receipt of the formal notifications required to be made to the local M.O.H. and by him passed on to the tuberculosis officer.

### Sanatorium Treatment.

The number of beds provided in hospitals and sanatoria approximates to the standard recommended by the Departmental Committee on Tuberculosis in 1912. The Council provide 800 beds for tuberculous patients of all classes. These beds are contained partly in sanatoria and hospitals belonging to the County Council and partly in institutions belonging to other local authorities or voluntary bodies. Altogether the Council have arrangements with some fifty-five separate institutions for the treatment of patients. To eliminate the waiting list and make really satisfactory provision for the treatment of non-pulmonary tuberculosis in children and adults, the county requires accommodation in the proportion of about one bed for 2,000 of population, or 875 beds. The county have in fact acquired two large mansions and estates, one of which is being converted into a pulmonary hospital for forty patients, and the other is intended as a special hospital for children as soon as financial exigencies permit.

### The Stay in the Sanatorium.

The length of stay in the Sanatorium depends on the recommendation of the medical superintendent. The average duration is three or four months. Recognition is given to the importance of making the patient's stay as interesting and free from monotony as possible. Graduated work is arranged and the patient's individual capacity and experience are studied in this connection. Poultry-keeping, carpentering, boot-making, etc., find favour. Medicines are discouraged. Week-end leave is granted. Lectures to the patients in small groups emphasise the educational character of the treatment and its importance after the patient returns home.

### A Unique Record.

Whither does all this lead ? This is a question being asked in connection with sanatorium treatment generally. What are the results ? In reply to this inquiry, Dr. Lissant Cox was able to produce very valuable data affording a comparison between the after-histories of patients who had and of those who had not undergone sanatorium treatment. As the result of the analysis of 3,948 histories of adults who began treatment during the five years 1914-18, so far as comparison is possible, it is found that patients in the early and intermediate stages of pulmonary tuberculosis who have undergone a course of sanatorium treatment, even for three or four months, fare appreciably better in health in later years than those who did not receive such form of treatment. Thus, of 981 sanatorium cases and 728 non-sanatorium cases in the early and intermediate stages with negative or absent sputum commencing treatment during the five years 1914-1918, 16 per cent. of sanatorium cases had died at end of 1922 ; 42.6 per cent. of non-sanatorium cases had died at end of 1922. Of the 1,286 sanatorium patients and 953 non-sanatorium patients with positive sputum, 66.2 per cent. of sanatorium cases had died at end of 1922 ; 84.8 per cent. of non-sanatorium cases had died at end of 1922. The difference is very considerable in the negative or absent sputum cases, and in positive cases 18.6 per cent. better after sanatorium treatment. If the sanatorium treatment and home treatment groups were scientifically comparable, it would appear that in certain age groups of the positive cases sanatorium treatment, merely regarded from the point of view of prolongation of life, is not much better than home treatment. It must never be forgotten, however, that from the more important side of prevention of the disease a stay in a sanatorium has, in the main, a definite educational result on the patient, his family, and his friends.

### Kill that Cow!

As to non-pulmonary tuberculosis, Dr. Cox pointed out that in the county there are over 1,000 children suffering from gland, bone, and joint tuberculosis, and the disease has doubtless been caused in half, or rather more than half, of these cases by milk from tuberculous cattle. The County Council is spending approximately £9,000 per annum on the institutional treatment of children with non-pulmonary tuberculosis. The amount for the whole country will be vastly greater. Would it not be better, asks Dr. Cox, to deal with one important source of infection —diseased cattle—rather than attempt to cure the children and adults after they have contracted the disease ? An interesting feature of the administration in connection with the treatment of children is that each of the tuberculosis officers in Lancashire has spent some time with Sir Henry Gauvain, at Alton, in Hampshire, studying the newest and most scientific methods of treatment of non-pulmonary tuberculosis.

### The Incidence of the Disease and its Elimination.

In conclusion, Dr. Lissant Cox said that recent investigation had established that cotton-spinning centres fared worse with tuberculosis than the weaving districts, and that the coal-mining areas—as was to be expected from the peculiar nature of the Lancashire coal dust—suffered more than the average for the county. Bad housing unquestionably aggravated the complaint. Do not, however, said Dr. Cox, expect that you are going to eliminate tuberculosis by remedying housing conditions alone. Bovine tuberculosis can be got rid of by a courageous handling of the milk question ; if that were accomplished and all infective cases from overcrowded homes isolated, the problem of the elimination of tuberculosis would be in a air way of solution.

## Figures and Tables

**Figure f1:**
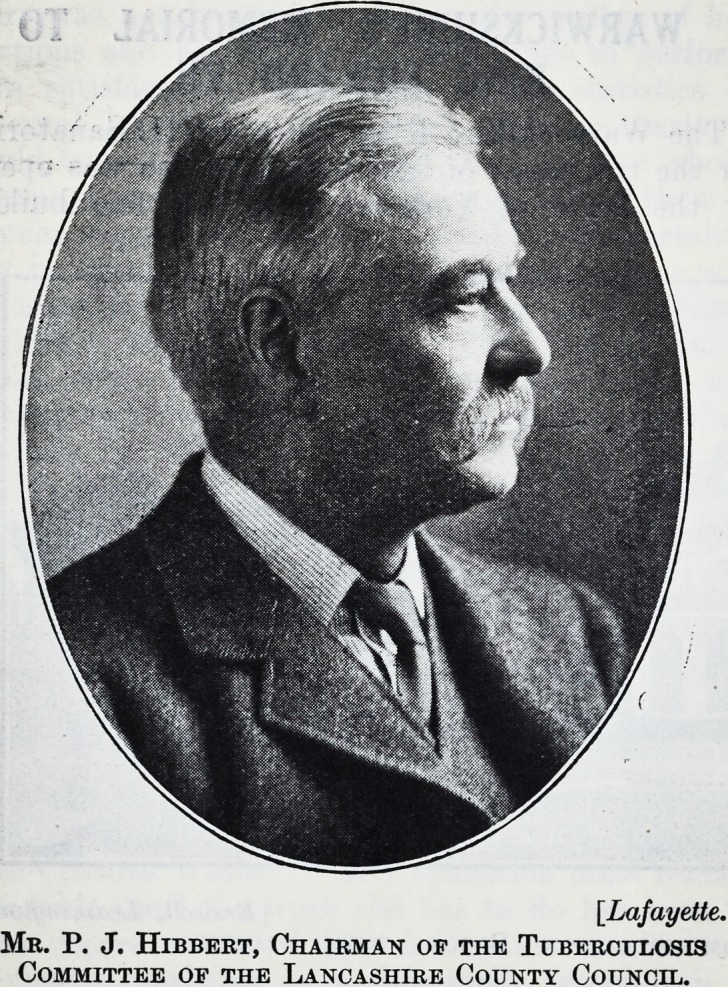


**Figure f2:**